# Impact of vaccination on COVID-19-associated admissions to critical care in England: a study of population linked data with quantitative bias analysis for linkage error.

**DOI:** 10.23889/ijpds.v7i3.1943

**Published:** 2022-08-25

**Authors:** David Harrison, Peter Watkinson, James Doidge, Manu Shankar-Hari, Paul Mouncey, Martina Patone, Carol Coupland, Julia Shankar-Hippisley-Cox, Kathryn Rowan

**Affiliations:** 1 Intensive Care National Audit and Research Centre; 2 University of Oxford; 3 NIHR Biomedical Research Centre, Oxford University Hospitals NHS Trust; 4 Intensive Care National Audit and Research Centre; 5 Centre for Inflammation Research, The University of Edinburgh; 6 Nuffield Department of Primary Health Care Sciences, University of Oxford; 7 School of Medicine, University of Nottingham

## Objectives

To explore associations between COVID-19 vaccination and admission to critical care with COVID-19 in England, by age group, dose, and over time.

## Approach

We conducted a population cohort study by linking the Case Mix Programme national clinical audit of adult intensive care to the National Immunisation Management System, combined with population denominators from the Office for National Statistics. We compared patient characteristics and estimated rates of critical care admission by vaccination status and age group and explored variation in admission rates and relative protection over time. Finally, we used quantitative bias analysis to explore sensitivity to potential linkage error.

## Results

Between 1 May and 15 December 2021 (England’s Delta wave), 11,431 patients were admitted to adult critical care units in England with COVID-19, of whom 10,141 were included in the cohort. Vaccinated patients were older, with higher levels of severe comorbidity and immunocompromise. Unvaccinated patients were greatly overrepresented, with people who had received two or more doses being at least 92% less likely to be admitted compared with those who were unvaccinated, for all ages, once the second dose had been rolled out. Those aged 50 years or older who had received a booster/third dose were 98-99% less likely to be admitted compared with unvaccinated. Our analysis was robust to linkage error, but closely related analyses could have been exquisitely sensitive to missed links.

## Conclusion

Our findings are consistent with a high degree of vaccine-acquired protection against critical care admission, with little waning in the first 6-9 months for those aged under 70. While our analysis was robust to linkage error, significant care is warranted with related analyses of linked vaccination and outcome databases.

